# In this month’s Bulletin

**DOI:** 10.2471/BLT.20.000720

**Published:** 2020-07-01

In the editorial section Haileyesus Getahun et al. (442) propose five measures to improve antimicrobial use and oversight during the COVID-19 pandemic response.

Tatum Anderson (445–446) reports on a feasibility trial in Liberia that provides hand-held ultrasound monitors to mothers in labour. Sabina Faiz Rashid talks to Andréia Azevedo Soares (447–448) about anthropology, poverty, inequality and sex education in Bangladesh.

## China 

### Antibiotics without a prescription 

Yanhong Gong et al. (449–457) estimate over-the-counter and online pharmacy sales.

### Lockdown effects 

Zheming Yuan et al. (484–494) model the impact of the February 2020 lockdown of Wuhan City.

## China, Indonesia, Kazakhstan, Malaysia, Philippines and Thailand

### Sufficient cancer treatment?

Alessandra Ferrario et al. (467–474) estimate sales volumes for chemotherapeutic agents.

## Finland

### Infection prevention in hospitals 

Helena Ojanperä et al. (475–483) study hand-hygiene compliance and nosocomial infections.

## Nigeria 

### Essential medicines 

Omotayo Fatokun (507–508) explains how regulatory changes are designed to increase local production.

## Global

### Paediatric oral antibiotics 

Grace Li et al. (458–466) find that most antibiotics are not sold in child-friendly formulations.

### Genetic variation in circulating SARS-CoV-2 

Takahiko Koyama et al. (495–504) analyse available sequence data. 

### Programme design for sexual and reproductive health and rights

Jessica D Gipson et al. (505–506) argue for the inclusion of infertility.

